# Effects of a music-visual guided physical activity promotion program for adults with intellectual disability in supported care settings: a cluster-randomized controlled trial

**DOI:** 10.1186/s12966-026-01872-6

**Published:** 2026-01-17

**Authors:** Ho Yu Cheng, Sek Ying Chair, Kai Chow Choi, Ka Ki Wong, Wai Leung Tang, Mohammed Usman Ali, Janet Wing Hung Sit, Erik Fung

**Affiliations:** 1 The Nethersole School of Nursing, Faculty of Medicine, The Chinese University of Hong Kong, Hong Kong, Hong Kong SAR China; 2 Hong Kong Anti-Cancer Society, Hong Kong, Hong Kong SAR China; 3 Martha Boss Lutheran Day Activity Centre, Hong Kong Lutheran Social Service, Hong Kong, Hong Kong SAR China; 4 Division of Life Science, The Hong Kong University of Science and Technology, Hong Kong, Hong Kong SAR China

**Keywords:** Functional exercise capacity, Intellectual disability, Exercise self-efficacy, Physical activity, Supported care settings

## Abstract

**Background:**

Adults with intellectual disabilities (ID) in supported care settings face significant cardiometabolic health disparities, exacerbated by barriers to physical activity (PA). Sustainable, theory-driven interventions are essential to address these challenges and promote cardiovascular health in this vulnerable population. This study evaluated the effectiveness of a novel music-visual guided PA intervention (MVgPA) in increasing moderate-to-vigorous PA time, improving self-efficacy and interest in PA, and enhancing functional exercise capacity among adults with ID in supported care settings. It also assessed the intervention’s acceptability among participants and care staff.

**Methods:**

In a 25-week cluster-randomized controlled trial, 16 supported care settings (*N* = 238 adults with mild-to-moderate ID) were randomized 1:1 to MVgPA (*n* = 8 settings, 120 participants; staff training plus twice-weekly 75-minute music-paced, visually cued PA sessions) or usual care (*n* = 8 settings, 118 participants). The Information-Motivation-Strategy (IMS)-based MVgPA intervention targeted knowledge, motivation, and practical strategies. The primary outcome was accelerometer-measured moderate-to-vigorous PA time. Secondary outcomes included functional exercise capacity (6-minute walk test), exercise self-efficacy (Baseline Interview Questionnaire), and PA interest (visual analogue scale). Outcomes were measured at baseline, 13, and 25 weeks, and analyzed using three-level mixed-effects models accounting for clustering, repeated measures, and individual variability.

**Results:**

No significant between-group differences emerged in moderate-to-vigorous PA time. Compared to controls, the MVgPA group showed significant improvements in functional exercise capacity at 13 weeks (Time*Group interaction coefficient, β: 20.89, 95% CI: 7.81 to 33.98, *p* = 0.002) and 25 weeks (β: 16.78, 95% CI: 2.44 to 31.12, *p* = 0.02); exercise self-efficacy at 13 weeks (β: 0.60, 95% CI: 0.10 to 1.10, *p* = 0.02) and 25 weeks (β: 0.96, 95% CI: 0.42 to 1.49, *p* < 0.001), and PA interest at 13 weeks (β: 1.22, 95% CI: 0.48 to1.96, *p* = 0.001) and 25 weeks (β:1.76, 95% CI: 0.98 to 2.55, *p* < 0.001). All intervention settings committed to sustaining MVgPA post-trial. The trial achieved 100% participant retention and demonstrated high acceptability (staff willingness scores: 4 to 6/6).

**Conclusions:**

The IMS-based MVgPA intervention significantly improved functional and psychosocial outcomes associated with cardiovascular health in adults with ID, despite no increase in objectively measured PA. The intervention’s high acceptability and sustainability support its scalable implementation in supported care settings.

**Trial registration:**

The study protocol was registered at ClinicalTrials.gov (NCT04938999) https://clinicaltrials.gov/study/NCT04938999?term=NCT04938999&rank=1 on 2021-06-25.

**Supplementary Information:**

The online version contains supplementary material available at 10.1186/s12966-026-01872-6.

## Background

Intellectual disability (ID), characterized by significant limitations in intellectual functioning and adaptive behavior, affects approximately 1.74% of the global population [1]. Adults with ID experience a higher prevalence of cardiometabolic diseases, including cardiovascular disease, diabetes, and hypertension, with earlier onset compared to the general population [2, 3]. These conditions contribute to substantial healthcare costs and reduced life expectancy, underscoring a critical global health disparity [4–6].

Inadequate physical activity (PA) is a key modifiable risk factor driving these outcomes. Sufficient PA enhances cardiovascular fitness, reduces cardiometabolic risk, and may lower dementia incidence by up to 18% in this population [7–9]. However, adults with ID face unique barriers to achieving recommended PA levels (≥ 150 min of moderate-intensity or 75 min of vigorous-intensity PA weekly), with 73% classified as physically inactive compared to 50% of the general population [10, 11]. Those in supported care settings, such as group homes or residential facilities, are particularly inactive due to intrinsic barriers (e.g., multi-morbidity, cognitive and behavioral challenges) and extrinsic barriers (e.g., limited access to exercise facilities, insufficient staff support) [12, 13].

The information-motivation-strategy (IMS) model, grounded in the Health Belief Model and Theory of Planned Behavior, provides a framework for promoting PA among adults with ID [14]. It emphasizes three determinants: information (understanding of adequate PA), motivation to perform PA, and strategy (practical approaches to sustain PA). Self-efficacy and social support from staff and peers are critical motivators for this population [15], with study showing a positive correlation between exercise self-efficacy and PA levels (*r* = 0.44, *p* < 0.05) [16]. The IMS model’s propositions align well with established facilitators of PA among adults with ID. At the personal level, key facilitators include peer social interaction, progress-contingent encouragement, and reward-based reinforcement [12, 13]. Environmental facilitators emphasize enjoyable program components, routine-based activities, and staff engagement in PA promotion [15]. This multi-level framework highlights the importance of addressing both individual and contextual factors to effectively promote sustained PA participation in this population.

Music-accompanied PA interventions have shown promising results in overcoming motivational barriers by increasing enjoyment, adherence, and exercise capacity [17, 18]. Karageorghis and Priest’s framework highlights music’s motivational properties through rhythm synchronization, cultural relevance, and reduced perceived exertion [19]. Pilot studies demonstrate that music-guided PA programs achieve high adherence (up to 90%) and improve functional capacity, supporting their potential for integration into supported care settings [17, 20].

The Music-Visual Guided PA (MVgPA) intervention, grounded in the IMS model, combines music-paced movement with visual cues to address both individual and environmental barriers. By targeting motivation and practical strategies, MVgPA intervention aims to enhance cardiovascular health outcomes and reduce global health disparities among adults with ID in supported care settings.

## Methods

### Aim and objectives

The primary aim of this study was to evaluate the effectiveness of a MVgPA intervention in increasing moderate-to-vigorous PA time (primary outcome), improving self-efficacy and interest in PA, and enhancing functional exercise capacity (secondary outcomes) among adults with ID in supported care settings. A second objective was to assess intervention’s acceptability and implementation using the RE-AIM framework.

### Study design

This prospective cluster-randomized controlled trial (ClinicalTrials.gov identifier: NCT04938999) was conducted from January 2021 to October 2023. The extended duration was necessary to address recruitment and implementation challenges during the coronavirus disease 2019 (COVID-19) pandemic. Modifications to the intervention protocol are described in the Intervention section. The RE-AIM framework (Reach, Effectiveness, Adoption, Implementation, Maintenance) guided the evaluation of the intervention’s effects and implementation outcomes.

### Participants and setting

Participants were recruited from 16 non-governmental supported care settings serving adults with ID in Hong Kong. These settings were identified from the public directory of residential services maintained by the Social Welfare Department in Hong Kong. A total of 20 eligible settings were approached using convenience sampling, of which 16 agreed to participate and were subsequently randomized. Eligibility criteria included: (1) Chinese adults aged 18–64 years with physician-confirmed mild-to-moderate ID; (2) capable of comprehending basic intervention instructions, as determined by care staff through a brief screening involving simple yes/no questions on following visual/auditory cues (e.g., stepping to music), with assisted decision-making permitted per institutional protocols, and ability to provide informed consent; (3) physically inactive (< 150 min of moderate-intensity or 75 min of vigorous-intensity PA/week, see Supplementary File 1); and (4) current residency in supported care settings.

To ensure participant safety, pre-participation health screenings followed the American College of Sports Medicine guidelines [21]. Medical clearance was required for participants with known cardiovascular, metabolic and/or renal disease, or related signs and symptoms (as detailed in Supplementary Table 1).

### Sample size, randomization and blinding

The study enrolled 238 participants across 16 supported care settings. Sample size was calculated based on a prior effect size of 0.41 (Cohen’s d) for PA improvements from a guided intervention in adults with ID [22]. This calculation provided 80% power at a two-sided 5% level of significance, accounting for an anticipated 20% attrition rate and a conservative intraclass correlation coefficient of 0.01. Based on these parameters, the required sample size was 238 participants across 16 clusters (119 per arm), which the study successfully achieved. Cluster-level randomization (at the care setting level) was employed to prevent cross-contamination between study arms. Baseline assessments was conducted before randomization, with settings allocated in a 1:1 ratio to the intervention (MVgPA) or control (usual care) groups. Participants from the same setting received the corresponding group assignment. An independent statistician generated the randomization sequence using a computer-based algorithm, with allocations determined by entry order. Group assignments were concealed from outcome assessors to minimize bias throughout the study.

### Intervention group

Supported care settings assigned to the intervention group implemented a standardized 12-week program consisting of twice-weekly 75-minute MVgPA sessions for participants with ID. This program was preceded by a preparatory training session for staff delivered one week before the start of the program. The MVgPA sessions incorporated synchronized auditory (music) and visual cues to enhance engagement and adherence, targeting moderate-intensity PA. The intervention’s conceptual framework and intervention plan are detailed in Supplementary Fig. 1 and Supplementary Table 2 .

#### IMS for staff: preparatory session and ongoing support

One week before the MVgPA sessions commenced, staff attended a 90-minute training led by a trained registered nurse (RN) and research assistant (RA1), based on the IMS model. The training covered: (1) Information - education on health benefits, safety, and moderate-intensity PA; (2) Motivation – identification of three positive MVgPA outcomes; and (3) Strategy - collaborative solutions for implementation barriers.

#### IMS for adults with ID: 12-week music–visual guided PA sessions

The MVgPA program promoted moderate-intensity PA (50–70% of maximal heart rate) through synchronized upper-limb and stepping exercises paced to music tempos (beats per minute [bpm]) (IMS: information, motivation, and strategy). A curated music library, encompassing children’s songs to popular music, was tailored to accommodate age diversity and the needs of individual with Down syndrome [18, 21–23], with tempo validated through pilot testing and heart rate monitoring to ensure safety and appropriate intensity. Group sessions (10–12 participants) were planned to be conducted twice weekly for 75 minutes, guided by a PowerPoint slideshow displaying actions. Each session included 5-minute warm-up and cool-down stretches, with 10-minute PA bouts synchronized to music. Staff and RA1 provided encouragement and positive reinforcement to enhance motivation (IMS-motivation). A refreshed MVgPA module (new exercise variations and music) was introduced at week 6 to sustain interest, without altering core protocol. The 24 sessions progressed as follows: the first six sessions were co-led by RA1 and care staff to support initial implementation and handover. An additional two sessions at week 6 were co-facilitated by RA1 and care staff to introduce the refreshed module. Care staff led the remaining sessions independently. Implementation reviews at weeks 6 and 9, involving the RN and RA1, reinforced progress, addressed concerns and optimized facilities using IMS strategies. The number of sessions conducted and participant attendance were recorded and reviewed by the RA1 and RN.

COVID-19 adaptations (as delivered): Pandemic restrictions varied throughout the study period, necessitating adaptations to ensure safety while maintaining protocol fidelity and equivalent participant exposure as far as possible. Group sizes were reduced to 2–4 participants for physical distancing, with sessions repeated as needed to provide the planned 24 sessions and total exposure time. During facility access restrictions, sessions were delivered virtually via a real-time online platform (Zoom). Delivery mode therefore varied across care settings depending on prevailing restrictions and timing of program implementation. On average, 5 of the 8 research staff co-facilitated sessions per setting were conducted online, although four facilities delivered all co-facilitated sessions in person.

### Control group

Participants in the control group were asked to maintain their usual daily activities and routines throughout the study period, with no group activities (consistent with pandemic-related restrictions that similarly applied to intervention settings), as documented by the research team at baseline and follow-ups. As an ethical consideration, these settings were offered the MVgPA program upon completion of all data collection, if interested.

### Data collection and outcome measurement

Outcomes were assessed at baseline, 13, and 25 weeks by a blinded research assistant (RA2) on-site at each supported care setting. Sociodemographic and clinical data were collected via participant interviews and record reviews.

The primary outcome, mean daily moderate-to-vigorous PA time, was measured using the validated Fitbit Charge 2, worn over five consecutive weekdays at each assessment time point (baseline, 13 weeks, and 25 weeks) [24, 25]. Participants wore devices during waking hours, except for water-based activities, with staff documenting wear time and any removals.

PA self-efficacy was assessed using the validated Chinese version of the Self-Efficacy Scale from the Baseline Interview Questionnaire (BIQ-C), a 5-item, 3-point Likert scale (1 = Not at all sure, 3 = Totally sure) [16]. The Cronbach’s alpha of this scale in the current study was 0.72.

Functional exercise capacity was evaluated using the six-minute walk test (6MWT), a validated and reliable measure for adults with ID [26]. Following standardized protocols, participants were instructed to walk as quickly as possible for six minutes along a 30-meter straight, flat, and hard surface under supervised conditions.

Program interest and satisfaction were measured via a 100-mm visual analogue scale (VAS), anchored by “no interest/not satisfied at all” (0 mm) and “most interested/most satisfied” (100 mm).

Staff satisfaction and willingness to sustain MVgPA were assessed at 3 months using a 6-point Likert scale (0 = not satisfied/willing at all; 5 = most satisfied/willing).

### RE-AIM framework indicators and targets

The RE-AIM framework was used to evaluate the intervention’s acceptability and implementation outcomes. Reach was measured by the proportion of eligible adults with ID who consented and completed MVgPA, relative to those approached. Effectiveness was assessed by comparing post-intervention changes between the intervention and control groups, with target including: (1) ≥ 0.35 standardized mean difference (SMD) increase in moderate-to-vigorous PA time [22]; (2) ≥ 0.5 SMD increase in PA self-efficacy (BIQ-C) and interest (VAS); and (3) ≥ 0.5 SMD increase in 6MWT distance. An SMD of 0.5 corresponds to a conventional medium effect size in behavioral and rehabilitation research. Participant and staff satisfaction levels were also evaluated to assess acceptability. Adoption was measured by the proportion of eligible care settings consenting to implement MVgPA. Implementation was assessed by the number of intervention sessions delivered per setting and participant attendance rates, with attendance recorded for all sessions. Maintenance was evaluated by the proportion of intervention settings intending to continue MVgPA and the number of sessions conducted during a 3-month follow-up period.

### Statistical analyses

Baseline characteristics were summarized using descriptive statistics. Normality was assessed via skewness, kurtosis, and normal probability plots; PA level required square-root transformation to meet assumptions. A three-level random intercept mixed-effects model (observations nested within participants nested within supported care settings) was used. Random intercepts for participants and care settings accounted for individual variability and clustering, respectively. A first-order autoregressive covariance structure was applied to level 1 residuals to model within-participant serial correlation over time (baseline, 13 weeks, 25 weeks). Time*Group interactions were tested to evaluate intervention effects, with significant positive coefficients indicating greater improvements in the intervention group at 13 and 25 weeks relative to baseline. Intention-to-treat analyses included all randomized participants, using restricted maximum likelihood estimation to handle missing data. Cohen’s d effect size was calculated from change scores at 13 and 25 weeks relative to baseline. All analyses were conducted in SAS 9.4 (PROC MIXED), with two-sided tests at α = 0.05.

## Results

### Characteristics of study participants

Of the 238 participants (mean age: 44.37 ± 10.17 years), approximately half were male, and had moderate levels of ID. The average residency duration was 11.22 years, with most (86.1%) employed in sheltered workshops. Hypertension (17.2%) was the most common comorbidity, and 12.2% had Down syndrome. Baseline characteristics were similar between the two groups (Table 1).

**Table 1:** Baseline characteristics of study participants

	Participants, No. (%) (*n* = 238)		
	Control Group (*n* = 118)	Intervention Group (*n* = 120)	Total
Sociodemographic characteristic			
Age, mean ± SD [range], y	43.20 ± 9.84 [21–64]	45.53 ± 10.41 [18–64]	44.37 ± 10.17 [18–64]
Sex, N (%)			
Male	55 (46.6)	51 (42.5)	106 (44.5)
Female	63 (53.4)	69 (57.5)	132 (55.5)
Level of ID, N (%)			
Mild	63 (53.4)	52 (43.3)	115 (48.3)
Moderate	55 (46.6)	68 (56.7)	123 (51.7)
Working in a shelter workshop, N (%)	105 (89.0)	100 (83.3)	205 (86.1)
Duration of stay in the residential care facility, mean ± SD [range], y	11.14 ± 8.75 [0.1–27]	11.29 ± 9.72 [0.1–31]	11.22 ± 9.23 [0.1–31]
Clinical characteristics, N (%)			
Hypertension	17 (14.4)	24 (20.0)	41 (17.2)
Hyperlipidemia	11 (9.3)	6 (5.0)	17 (7.1)
Diabetes	13 (11.0)	10 (8.3)	23 (9.7)
Epilepsy	11 (9.3)	12 (10.0)	23 (9.7)
Autistic spectrum disorder	8 (6.8)	4 (3.3)	12 (5.0)
Down syndrome	15 (12.7)	14 (11.7)	29 (12.2)
Other mental illness	12 (10.2)	18 (15.0)	30 (12.6)

*ID* Intellectual disability, *SD* Standard deviation

### Effectiveness

Table 1 presents the means and standard deviations for the study outcomes at each time point and the effects of the MVgPA intervention relative to the control group (estimated by the mixed-effects model) and corresponding effect sizes.

**Table 2:** The between-group comparison of outcome variables across the study time points by mixed-effects models

Outcomes	Timepoint	Intervention Group (*n* = 120)	Control Group (*n* = 118)	Time*Group Interaction effect	Effect size	
		**Mean** ± **SD**	**Mean** ± **SD**	**β (95% CI)**	**Cohen’s d (95%CI)**	***P*** **Value**
PA time*	Baseline	13.67 ± 23.70	7.73 ± 22.00			
	T1	10.53 ± 21.84	4.00 ± 11.05	-0.02 (-0.56 to 0.52)	--	0.94
	T2	12.78 ± 26.63	6.29 ± 18.45	0.11 (-0.56 to 0.78)	--	0.77
PA self-efficacy	Baseline	12.08 ± 2.59	13.29 ± 1.99			
	T1	13.17 ± 2.17	13.70 ± 1.70	0.60 (0.10 to 1.10)	0.26 (0.004 to 0.52)	*0.02*
	T2	13.42 ± 2.01	13.71 ± 1.72	0.96 (0.42 to 1.49)	0.45 (0.19 to 0.71)	*< 0.001*
Functional exercise capacity (6MWT)	Baseline	259.69 ± 80.23	268.22 ± 80.38			
	T1	289.48 ± 80.18	274.4 ± 77.60	20.89 (7.81 to 33.98)	0.41 (0.15 to 0.67)	*0.002*
	T2	280.09 ± 74.23	271.61 ± 75.12	16.78 (2.44 to 31.12)	0.31 (0.05 to 0.57)	*0.02*
Interest in performing PA	Baseline	7.58 ± 2.94	8.53 ± 2.60			
	T1	8.81 ± 2.14	8.53 ± 2.91	1.22 (0.48 to 1.96)	0.42 (0.16 to 0.68)	*0.001*
	T2	9.09 ± 2.26	8.29 ± 3.10	1.76 (0.98 to 2.55)	0.58 (0.32 to 0.84)	*< 0.001*

*SMD* Standardized mean difference, *6MWT* Six-minute walk test, *T1* Immediately post-intervention, *T2* 3-month follow up

^*^PA time was measured by the minutes of moderate-to-vigorous intensity exercise/day. The value of PA time was transformed by square root when analyzed using the mixed-effects model

Comparing with the control group, the intervention group showed no significant improvements in moderate-to-vigorous PA time across the study time points.

Among the secondary outcomes, the intervention group showed significantly greater improvement in PA self-efficacy versus control group at 13 weeks (Time*Group interaction coefficient, β: 0.60, 95% CI: 0.10 to 1.10, *p* = 0.02) with a small effect size (Cohen’s d = 0.26) and at 25 weeks (β: 0.96, 95% CI: 0.42 to 1.49, *p* < 0.001) with a small-to-moderate effect size (d = 0.45). The intervention group also showed greater improvement in functional exercise capacity compared with controls at 13 weeks (β: 20.89, 95% CI: 7.81 to 33.98, *p* = 0.002) with a small-to-moderate effect size (d = 0.41), and at 25 weeks (β: 16.78, 95% CI: 2.44 to 31.12, *p* = 0.02) with small-to-moderate effect size (d = 0.31). Additionally, the intervention group showed significantly greater improvements in PA interest compared with controls at 13 weeks (β: 1.22, 95% CI: 0.48 to 1.96, *p* = 0.001) with a small-to-moderate effect size (d = 0.42) and at 25 weeks (β: 1.76, 95% CI: 0.98 to 2.55, *p* < 0.001) with a moderate effect size (d = 0.58).

Regarding satisfaction of the MVgPA program, participants in the intervention group (*n* = 120) reported high levels (mean 9.09 ± 1.94 out of 10). Staff (*n* = 17 across 8 facilities), comprising welfare workers (47.1%), residential instructors (41.2%), and administrative staff (17.6%), reported strong satisfaction (mean 5.12 ± 0.49 out of 6). Staff respondents had an average of 4.4 ± 4.51 years of service experience, reflecting diverse perspectives. Overall, these findings also indicated the MVgPA was well accepted by adults with ID and staff.

### Reach and adoption

**Fig. 1:**
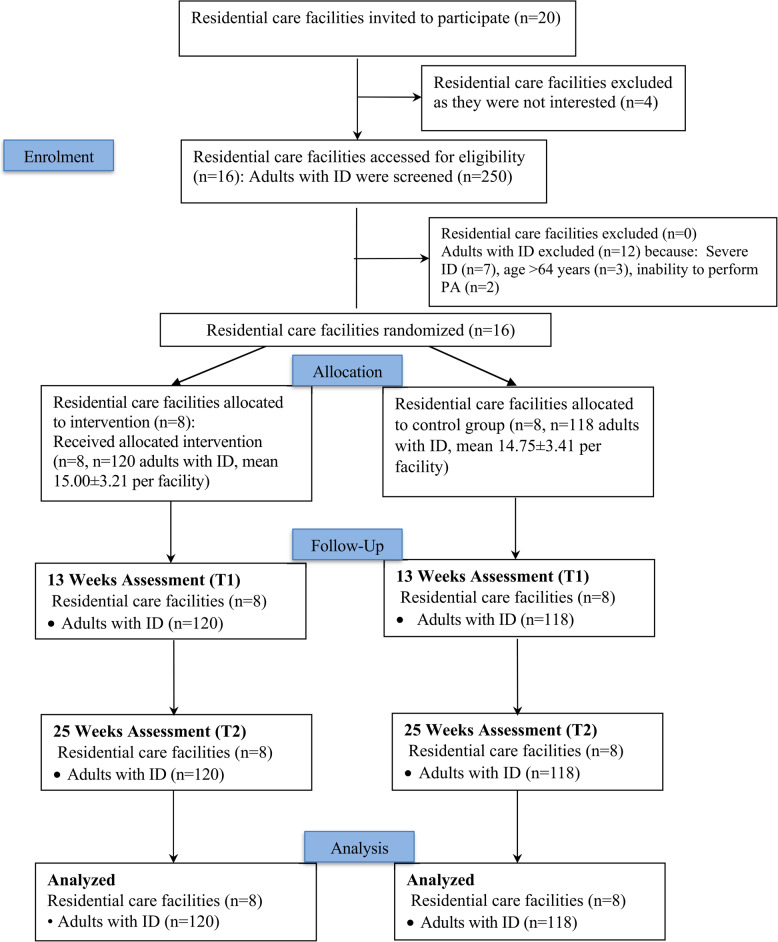
Trial flow diagram

Of 20 supported care settings contacted, 16 participated (80% adoption rate) and were randomized equally to intervention (*n* = 8) or control (*n* = 8) groups. Among 250 adults with ID approached, 238 were eligible and enrolled (95.2% reach; exclusions: *n* = 7 for severe ID, *n* = 3 for age > 64 years, *n* = 2 for inability to perform PA). Incomplete data rates were low (1.2–3.5%), attributable to participant refusal to complete questionnaires, perform the 6MWT, or wear activity trackers despite staff encouragement. The participant flow is illustrated in Fig. 1.

### Implementation

The intervention program comprised 24 MVgPA sessions over 12 weeks. Eight sessions were co-facilitated by research and care staff, and 16 were led by care staff alone. Staff adherence was high, with 87.5% completion of co-facilitated sessions and 75% of independent sessions, as monitored via session logs and RA1 reviews. Variations were primarily due to COVID-19 disruption (e.g., 25% cancellations), but IMS-based reviews at weeks 6 and 9 ensured protocol fidelity > 80% across all 8 settings. Participant attendance averaged 83.4% (SD = 6.2%), with absence due to COVID-19 infections, scheduled home leave, or behavioral challenges (e.g., temper tantrums).

### Maintenance

All intervention settings (*n* = 8) expressed intent to continue MVgPA (willingness scores: 4–6 out of 6) post-trial and independently delivered an average of 15 sessions during the 3-month follow-up period. Additionally, three settings expressed interest in promoting the MVgPA to other care settings.

## Discussion

This study developed and evaluated a novel interactive MVgPA program designed to overcome personal and environmental barriers to PA initiation and maintenance among adults with ID in residential care settings. Implemented during multiple COVID-19 waves, the intervention was adaptively delivered through modified group sizes and a hybrid in-person/online format to comply with evolving infection control measures. The MVgPA program demonstrated strong acceptability among both participants and facility staff, while yielding significant improvements in three key domains: functional exercise capacity, PA self-efficacy and interest in PA. However, no significant between-group differences were detected in changes to objectively measured PA time.

The MVgPA program demonstrated strong acceptability, evidenced by an 80% facility participation rate (16/20 approached) and 95.2% individual-level recruitment success (238/250 eligible adults with ID). High engagement was also reflected in good retention (100% completion of study) and attendance (83.4%). These implementation metrics—superior to those typically reported in physical activity interventions for adults with ID—may have been facilitated by the program’s design, which was tailored to known barriers in this population (e.g., cognitive and motivational challenges) based on prior literature [13, 23]. Additionally, the study was conducted during a period of COVID-19 restrictions when physical inactivity was a recognized challenge for residents with ID [27–29]. The observed recruitment and retention success suggests the program was perceived as relevant in this context, though direct perceptions of staff or caregivers were not formally assessed. Using the RE-AIM framework, the MVgPA intervention exceeded targets for Reach (95.2% vs. ≥80%), Adoption (80% vs. ≥70%), Implementation (81.3% delivery and 83.4% attendance vs. ≥80% and ≥ 75%), and Maintenance (100% intent and mean 15 sessions vs. ≥70% and ≥ 10), demonstrating strong potential for real-world scalability. Effectiveness targets were partially met for secondary outcomes (SMD 0.26–0.58 vs. ≥0.5), with the primary outcome falling short amid pandemic disruptions, underscoring the need for adaptive designs in crisis contexts.

The MVgPA protocol was developed in close collaboration with co-investigators possessing extensive residential care expertise, ensuring pre-pandemic feasibility for routine implementation in care facilities. Subsequent pandemic-specific adaptations to infection control protocols preserved high engagement, as evidenced by an overall attendance rate of 83.4%. Furthermore, all intervention settings reported high willingness to continue the program post-trial, with independent delivery of an average of 15 sessions during the 3-month follow-up period. These findings suggest good acceptability and low perceived ongoing burden among staff, consistent with the IMS framework’s emphasis on staff empowerment and sustainable strategies. However, no significant between-group differences in moderate-to-vigorous PA change were found at follow-up, with both groups showing comparable declines post-intervention. This pattern is consistent with broader evidence that pandemic-related restrictions (e.g., quarantine and social distancing measures) limited opportunities for physical activity among adults with ID in residential settings [27, 29].

Furthermore, the MVgPA program demonstrated significant psychosocial and functional benefits for adults with ID, yielding small-to-medium effect size improvements in PA self-efficacy, functional exercise capacity, and PA interest that were sustained at 3-month follow-up. These outcomes were achieved through carefully designed IMS model-based strategies implemented at both staff and participant levels. For facility staff, the intervention incorporated: (1) preparatory workshops addressing anticipated implementation barriers, (2) hands-on co-facilitation opportunities with research team support, and (3) ongoing capacity-building to sustain organizational PA promotion. For participants, the program utilized music-based pacing with familiar, preferred selections to both enhance self-efficacy (through rhythmic cueing) and ensure moderate-to-vigorous intensity targets were met. Staff feedback highlighted exceptional participant engagement, with most facilities documenting spontaneous participant-initiated reminders about upcoming sessions, which persisted even post-intervention. This high intrinsic motivation was further evidenced by high satisfaction scores across program components, suggesting the MVgPA approach successfully addressed core behavioral determinants of PA participation in this population.

A recent systematic review of nine studies (five specifically targeting adults with ID) demonstrated that PA interventions tailored to residents’ daily routines can yield short-term improvements in PA levels [30]. Similarly, another systematic review of six PA promotion programs for this population found that group-based PA education with active participation led to short-term increases in self-reported weekly PA [31]. Furthermore, a separate systematic review revealed that lifestyle interventions for adults with ID frequently incorporate specific behavior change techniques, including provision of health information, social support planning, behavioural instruction and goal setting [23]. The current findings align with pre-pandemic meta-analytic evidence demonstrating consistent exercise intervention effects on self-efficacy and functional capacity in adults with ID [7, 22], while extending these benefits to pandemic conditions. Notably, prior research has established functional capacity as a robust predictor of both light- and moderate-to-vigorous intensity PA in residential settings, independent of demographic covariates (age, sex, and body mass index) [32]. In the current study, significant improvements were observed in participants’ PA self-efficacy and functional exercise capacity, but no significant between-group differences in moderate-to-vigorous intensity PA minutes. This pattern of findings suggests that participants in the MVgPA intervention may have increased their light-intensity PA, while pandemic-related restrictions hindered their abilities to achieve the desired moderate-to-vigorous intensity PA. Pandemic restrictions (e.g., onsite quarantines, social distancing) likely constrained MVPA gains, as both groups showed similar post-intervention declines despite sustained psychosocial benefits (self-efficacy, functional capacity, PA interest) in the intervention arm. This pattern mirrors broader evidence of sharp PA reductions in adults with ID during COVID-19 due to facility restrictions and limited group activity [29, 33]. PA programs for residential ID populations should incorporate: (1) individualized moderate-intensity activities, (2) remote coaching for independent participation, and (3) adapted group formats compliant with distancing requirements. These strategies could maintain PA engagement during public health restrictions while addressing the current intervention’s limited impact on moderate-to-vigorous PA levels.

### Implications

This study demonstrates the IMS model’s efficacy in developing PA programs for adults with ID in residential care. Despite pandemic challenges, our intervention significantly improved self-efficacy, exercise interest, and functional capacity. Future research should validate the IMS framework across diverse settings, examine long-term maintenance, and assess impacts on varying PA intensities. For healthcare and social care professionals, the MVgPA program offers a practical, scalable solution requiring minimal staff training. Technology-enhanced adaptations could improve program resilience during health crises. At the policy level, integrating flexible PA programs into standard care and mandating systematic PA monitoring would enhance service quality. These measures align with public health priorities to reduce inactivity and prevent NCDs. Our findings underscore the importance of organizational capacity building and adaptable interventions to promote sustainable PA participation in this vulnerable population. Future programs should retain measurement of self-efficacy and 6MWT for their sensitivity to intervention effects, while adding light-PA accelerometry to capture broader activity patterns and qualitative enjoyment evaluations to inform engagement strategies, ensuring comprehensive evaluation of scalability and real-world impact.

### Study limitations

This study has several important limitations that should be considered when interpreting the results. First, the use of convenience sampling for facility recruitment. Specifically, approaching 20 eligible non-governmental supported care settings identified from a public directory, of which 16 agreed to participate, may have introduced selection bias, as participating facilities were likely more motivated to promote PA than non-participating ones. Second, although the intervention maintained the planned 12-week program structure with 24 sessions and equivalent total exposure time (achieved through reduced group sizes of 2–4 participants and session repetition), COVID-19 restrictions led to an extended calendar implementation period in several facilities and required virtual delivery for a substantial proportion of sessions. These adaptations may have reduced intervention density (sessions per calendar week) and diminished the social interaction and motivational benefits inherent in larger in-person group activities, potentially attenuating effects on behavioral and social outcomes. Third, the evaluation timeframe was limited to immediate post-intervention and 3-month follow-up assessments, leaving the longer-term sustainability of effects unknown. Finally, although objective PA measurement was employed, the failure to examine time spent at different intensity levels represents a significant gap, particularly regarding potential changes in light-intensity activity. Consequently, while the program demonstrated benefits for functional exercise capacity, we cannot determine whether it meaningfully influenced overall PA patterns, especially at lower intensities, among this population of adults with ID in residential care. Self-report measures (BIQ-C, VAS) proved feasible in this sample, as evidenced by 100% retention and low missing data (1.2–3.5%), due to one-on-one administration by a trained, blinded RA, use of visual aids (pictorial response cards), and simplified phrasing in a quiet, familiar setting. While cognitive and communication barriers in adults with ID necessitate caution in interpretation, internal consistency (Cronbach’s α = 0.72 for BIQ-C) and high engagement suggest acceptable reliability. Future studies should pair self-reports with proxy ratings or tablet-based tools to further enhance validity and inclusivity.

## Conclusions

This cluster-randomized trial evaluated an IMS-based MVgPA intervention for adults with ID in residential care during COVID-19. Despite pandemic challenges, the program achieved high adherence and acceptability, demonstrating feasibility during public health crises. Results showed significant improvements in PA self-efficacy, exercise interest, and functional capacity, although no significant change in moderate-to-vigorous PA time was observed. These findings highlight the intervention’s value for enhancing psychosocial and functional outcomes in residential settings, even under restrictive conditions. Future research should examine: (1) long-term maintenance effects, (2) dose-response relationships across PA intensities, and (3) strategies to translate motivational gains into increased PA participation. The program’s adaptability and minimal resource requirements support its scalability for this vulnerable population.

## Supplementary Information

Supplementary Material 1.

Supplementary Material 2.

Supplementary Material 3.

Supplementary Material 4.

## Data Availability

The datasets generated and analyzed during the current study are not publicly available due to privacy protection and ethical considerations but are available from the corresponding author on reasonable request.
