# Supplementary material for: Effects of a music-visual guided physical activity promotion program for adults with intellectual disability in supported care settings: a cluster-randomized controlled trial
Source: Int J Behav Nutr Phys Act. 2026 Jan 17;23:11. doi: 10.1186/s12966-026-01872-6 (PMC12896249; doi:10.1186/s12966-026-01872-6)
Supplement: Supplementary file 2 — Supplementary Material 2. [file 12966_2026_1872_MOESM2_ESM.docx]

**Study Protocol**

**Effects of a music–visual guided physical activity promotion program among adults with intellectual disability living in residential care facilities: A cluster-randomized controlled trial**

|  | Justification for conducting the project: | |
| --- | --- | --- |
|  |  | Health needs of the local community (supported by published data or relevant experiences) |
|  |  | The increased life expectancy of individuals with intellectual disability (ID) is accompanied with rising incidence of early onset age-related health problems. A local study revealed that adults with ID living in residential care facilities are at higher risk of cardiometabolic disease and earlier onset of dementia upon the age of 40 when compared to general population^1^. ID is defined as a significant limitation of intellectual functioning and adaptive behaviour, including practical and social skills, and with onset before the age of 18; its prevalence in Hong Kong is 1.4%^2^. The twofold increased risk entails a high-expense healthcare (>USD$ 4600 per user) due to high morbidity rates caused by non-communicable diseases among adults with ID at younger age^3^.Thus, a health promotion strategy is urgently needed in this specific population.  Apart from the genetic determinants and the influence of psychotropic medication on health, many of the health issues faced by adults with ID are related to inadequate physical activity (PA)^4^. Substantial evidence showed that adequate PA for adults with ID could improve their physical fitness, prevent cardiometabolic and mental diseases, and reduce the risk of dementia by 18%^5,6^. However, promoting adequate PA (≥150-min moderate-intensity or 75-min vigorous-intensity PA/week) to this population (US Department of Health and Human Service) is a major challenge worldwide, and Hong Kong is no exception. Adults with ID have higher prevalence of being physically inactive (73%)^7^ than the general population (50%)^8^. The situation is much worse among adults with ID living in residential care facilitates compared to those living with families^7^. A local study showed that adults with ID in residential care facilities only engaged 2% of daily time (10 min) in moderate-to-vigorous PA as measured by activity trackers^9^.  Most adults with ID tend to avoid PA^10^. Multi-morbidity, cognitive and behavioural impairments are the common intrinsic PA barriers of this population. Moreover, adults with ID also face external PA barriers, such as lack of access to exercise facilities, finances and support from staff of residential care facilities^11^. Given that one tenth of adults with ID live in 127 residential care facilities in Hong Kong, an appropriate and acceptable PA promotion intervention must be developed for this population experiencing health disparities. |
|  |  | Literature review on others’ strategies to address these needs |
|  |  | Even though the health benefits of PA have been widely studied^6^, only a few studies have investigated the initiation and maintenance of PA among adults with ID. A recent systematic review on PA promotion program for adults with ID showed that group-based PA education with participation could only improve the self-reported weekly PA level in the short term^12^. Another systematic review showed that lifestyle intervention for adults with ID tend to adopt certain behavioural change techniques, such as providing information, planning social support (which helps them achieve a certain behaviour), providing instructions on how to perform a certain behaviour, and setting goal^13^. However, in most PA interventional studies on adults with ID, the selection of these techniques is not theory driven^13^.  In addition, the unsustainable effects of a PA program can also be attributed to the limited environmental facilitator, that is, residential facilities have limited organizational capacity to promote PA of adults with ID^4^. The limited resources, time and knowledge of the staff hinder the promotion of health of adults with ID^4^. Therefore, behavioural change must be achieved at both the organizational and individual levels. |
|  |  | Scientific evidence supporting the strategies to address these needs proposed in this project |
|  | Basing on the health belief model and theory of planned behaviour, the information–motivation–strategy (IMS) model posits that the initiation and maintenance of PA among adults with ID are determined by three elements, **information** (understand what they are supposed to do for adequate PA), **motivation** to perform PA, and practical and appropriate **strategy** to maintain adequate PA^14^. In terms of motivation, the belief of being capable of performing PA (self-efficacy), as well as the social support from staff and others on PA participation are important for adults with ID^10^. A local study of adults with ID showed that exercise self-efficacy is significantly associated with PA level (r=0.44, p <0.05)^15^. The propositions of the IMS model are also consistent with the findings on the facilitator of PA among adults with ID. Specifically, personal facilitators include social interaction with peers, encouragements contingent on efforts and/or progress towards behaviour and praises/reward contingent on successful behaviour^13^, whereas environmental facilitators include fun components and regular nature of PA, as well as staff interest in PA^10^.  Music induces interest and enjoyment. Previous studies showed that PA with music increases the motivation and fun, and reduce the perceived discomfort of PA, resulting in a high PA completion rate (90%) and significant improvement in exercise capacity among adults with ID^16,17^. The rhythmical elements, melodic and harmonic aspects, cultural impact and the extra-musical association of music are the four factors of the motivational qualities of music in the PA context, in which the rhythm response is the most important^18^. The rhythm response refers to an innate human predisposition to synchronize movement with musical rhythms. Apart from serving as an information and motivation of PA, the music tempo is also a practical strategy for both adults with ID and the staff of residential care facilities to achieve moderate-intensity PA. The enhanced music tempo can be an audio reinforcement on the required pace of moderate-intensity PA, which is an important modality to maintain the compliance to PA among adults with ID^19^. We have conducted a four-week group-based music–visual guided PA (MVgPA) for adults with ID living in two hostels of the Hong Chi Association (n = 38) and found a high completion rate (90%) among the participants, a significant increase (15.5%) in PA enjoyment and a significant improvement in their functional exercise capacity as measured by six-minute walk test (23.6±40.9m, p<0.01). The staff of these facilities also reported that the MVgPA can be easily incorporated into the routine of their hostels and they will continue to implement this MVgPA. They were pleased with the improvement shown by their service users after the implementation of such intervention, thereby motivating them to promote PA in the facilities^5^.  PA offers cardiometabolic and psychological benefits to adults with ID. However, its initiation and maintenance pose considerable challenges to healthcare and social workers who support this vulnerable group. Facing the challenges of rising morbidity rates caused by non-communicable diseases among adults with ID at younger age, a timely development of an effective and sustainable strategy based on the IMS model is needed to enhance the PA level, PA self-efficacy and interest, and functional exercise capacity of adults with ID. | |
|  | Aim and Objectives: | |
|  | This project aims to develop a MVgPA intervention based on the IMS model to promote PA among adults with ID who are living in residential care facilities. The specific objectives of this project are to (1) evaluate the effectiveness of this intervention in improving the PA level of these adults, enhancing their self-efficacy and interest in performing PA and improving their functional exercise capacity by conducting a cluster-randomized controlled trial, and (2) examine the acceptability of this intervention among the participants and the staff of residential care facilities. | |
|  | Project Plan: | |
|  | (i) | Target group |
|  |  | ***Participant recruitment***  The person-in-charge of residential care facilities and the non-governmental organizations that serve adults with ID, including the Hong Chi Association who had collaborated with us in our pilot project, will be approached by the research team to seek their collaboration and support in recruiting participants and implementing the project. The research team will organize promotion talks and distribute promotion leaflets of the project to potential participants and their families or guardians. To ensure safety of the participants, PA pre-participation health screening will be performed in accordance with the guidelines of American College Sport Medicine (ACSM, Appendix 1). Medical clearance/approval to participate MVgPA will be sought if they have known cardiovascular, metabolic and/or renal disease, or signs and symptoms suggesting these diseases (as detailed in Appendix 1). An information sheet related to the MVgPA, including the heart rates during the PA and type of exercise, will be provided to the physicians. The eligibility of the participants will be screened based on the following criteria:  *Inclusion criteria:*   1. Chinese adults who are aged 18 years to 64 years and are diagnosed with mild or moderate ID based on the information provided by the residential care facilities; 2. Able to understand basic information and instructions of the intervention and to make decisions upon their participation; 3. Classified as physically inactive (i.e., <150-min moderate-intensity or 75-min vigorous-intensity PA/week, see Appendix 1); 4. Currently using residential service user as stated in the Social Welfare Department (https://www.swd.gov.hk/en/index/site_pubsvc/page_rehab/sub_listofserv/id_sermentalhandi/).   *Exclusion criteria:*   1. Currently enrolled in a regular exercise program; 2. Physically unable to participate in the MVgPA, such as having significant mobility problem; 3. Participation to MVgPA will put them at physiological risk; or 4. At risk of self-harm as determined by the staff of the residential care facilities.   ***Sample size calculation***  A similar study for adults with ID in community residences showed an effect size of 0.41 on PA level change^20^. Assuming a similar effect size on PA level change in our proposed trial, a sample size of 119 participants per each of the control and intervention groups will provide the study with 80% power at 2-sided 5% level of significance, as estimated using the power analysis software PASS 13.0 (NCSS, Kaysville, USA). Furthermore, to account for potential reduction in statistical efficiency due to randomization by residential care facilities (clusters) instead of individual participants, a variance inflation factor, called design effect, will be applied to impose to the estimated sample size. The design effect is given by 1 + (m–1)*ICC, where m=average cluster size and ICC=intracluster correlation coefficient of the underlying outcome. We anticipate that the outcomes of the participants recruited from the same residential care facility are unlikely correlated with one another, particularly for the PA outcome. In this regard, the ICC would be small. A synthesis study revealed that ICC tends to be small in primary care research with a median of 0.005 and inter-quartile range of 0.000 to 0.021^21^. Allowing for a conservative ICC of 0.01 and up to 20% attrition rate in our proposed study, at least 17 participants per each of the 14 residential care facility are therefore required.  ***Cluster randomization***  Randomization will be performed at the cluster level (residential care facility) instead of individual participants to avoid contamination between the intervention and control groups. Baseline data for all participants in each cluster will be completed before randomization. The facilities will be randomized to either intervention or control group in a 1:1 ratio. Eligible participants recruited from the same facility will be allocated to either intervention or control groups accordingly. Group allocation will be concealed from outcome assessors and made according to the random group allocation sequence generated by an independent statistician and the sequence of entry into the study. |
|  | (ii) | Implementation plan |
|  |  | **Preparation of the MVgPA**  The MVgPA aims to help adults with ID perform adequate moderate-intensity PA. According to the recommendation of ACSM^22^, the targeted heart rate for adults with ID who are engaged in moderate-intensity PA is 50% to 70% of maximal heart rate, which is determined not only by age but also by whether they have Down syndrome or not. Specifically, the maximal heart rate is calculated as [210 – 0.56*(age) – 15.5*(DS)], where DS is equal to 2 if the participant has Down syndrome and equal to 1 if the participant does not have Down syndrome^22^.  The MVgPA comprises upper limbs movement and stepping exercise. The pace of MVgPA is synchronised with the enhanced music tempo (bpm). With reference to Finalyson et al. study^23^, the pace of ≥109 steps/min is regarded as moderate-intensity PA. In consideration of the participants’ conditions and music preferences, a list of music with bpm ranges from 90 – 109 (below average), 110 – 129 (average) and 130 to 149 (above average)^24^, as well as ranges from children song to pop song will be prepared.  A local survey of 11,452 individuals with ID showed that the median age of residential service users ranged from 42.7 to 45.8 years with interquartile range of 32.8 to 53.4 years^25^. With age-related changes are observed since the age of 40 among adults with ID^25^, twenty adults with ID from age groups of 18 – 39 years and 40 – 64 year and with/without Down syndrome will be invited to perform MVgPA using these different sets of music tempo. Their heart rate will be monitored before and during the MVgPA to identify the sets of music tempo that can reach 50% to 70% maximal heart rate. Thus, a selection guide for appropriate ranges of music tempo will be developed based on the characteristics of these adults (i.e., age and Down syndrome) to achieve moderate-intensity level of PA.  **Delivery of the intervention**  Intervention group  The MVgPA adopts the IMS model to address the personal and environmental challenges in PA initiation and maintenance of adults with ID. This 13-week program includes a preparatory session for the staff and 12-week structured MVgPA sessions (Appendix 2 and 3).  **IMS for staff: preparatory session and ongoing support**  As discussed before, the knowledge and motivation of the staff are environmental facilitators of PA among adults with ID^10^. At one week before the structured PA session, a 90-min education on strategies to achieve adequate PA, including its associated health benefits and the safety concerns related to moderate-intensity PA, will be delivered to the staff by a trained registered nurse (RN) ***(IMS-information)***. The staff will then be asked to determine the three most significant obstacles that they expect to encounter when supporting their service users in performing adequate PA. As the resistance is explored, RN will facilitate the staff to devise specific solutions to these obstacles ***(IMS-strategy)***. Given that the staff will be co-facilitator of the MVgPA program, they will be asked to practice the MVgPA program with RN and a trained research assistant (RA1) as experiential learning. They will then identify the three positive outcomes of the MVgPA program on their service users to increase their motivation in implementing the MVgPA ***(IMS-motivation).***  To support the staff of residential care facilities in implementing 24 sessions of MVgPA in 12 weeks, the first six sessions will be led by the RA1 and co-facilitated by the staff, while the subsequent sessions will be conducted by the staff only. At week 6, another set of MVgPA will be implemented to increase the participant’s interest; two sessions will be co-facilitated by RA1. At weeks 6 and 9, the RN and RA1 will hold a meeting with the staff of the residential care facilities to acknowledge the achievement of the staff and adults with ID, as well as to address any of their concerns related to the implementation of MVgPA, and to identify the facilitators and tackle barriers to the implementation of the MVgPA (***IMS-strategies and motivation***). The number of session conducted and the attendance of participants will be recorded and reviewed by the RA1 and RN.   1. **IMS for adults with ID: 12-week music–visual guided PA session**   The structured MVgPA group session (10 – 12 participants) will be conducted twice per week. The 75-min music-paced physical activities will be presented in the PowerPoint slideshow. The instructions for an upper limb exercise will be presented in a large number pad in the slideshow, and the participants will then be asked to follow the actions and directions presented on the slide. The number pad exercise is adapted from the square-stepping exercise, which is a mind-motor activity^26^. Body movement that enhance peer interaction, such as clapping hands with the other participants, will be included. The participants will also be asked to perform stepping exercises along with the music tempo. Five-minute warm up and cool down exercises for stretching the major muscle–tendon units of the shoulder, body trunk, and limbs will be performed before and after the session. The use of music and number pad exercise aims to provide real-time PA information and strategies as well as enhance the motivation and interest of adults with ID in moderate-intensity PA **(*IMS-information, motivation and strategy*)**.  The music-paced PA will be scheduled in a 10-min bout. At the beginning of the 10-min bout, the participants will be oriented about the health benefit of adequate PA. During the 10-min bout of PA, both the staff and RA1 will provide the participants with words of encouragement, such as “you can do it!” and “You are doing great!”. Before the end of each bout, the participants will be asked to give themselves a “like”, as shown in the PowerPoint slide. After completing each bout, both the staff and RA1 will acknowledge the achievement of participants to enhance their confidence in their PA performance **(*IMS-motivation*)**. In addition, the mastery and vicarious experiences of being capable to perform the 10-min bout by self and peers, as well as the verbal persuasion from staff could enhance the PA self-efficacy of adults with ID. At week 6, another set of MVgPA will be implemented to increase the participant’s interest (***IMS-motivation***).  Before the implementation of the 12-week music-visual guided PA, a pilot group session will be conducted to finalize the music tempo for the MVgPA. The music tempo that is suitable for majority of the group members will first be chosen from the developed selection guide. Group members will then perform the MVgPA while their heart rates are monitored. The tempo of the music will be adjusted to ensure that all members achieve their heart rate to 50% to 70% of their maximal heart rate during the MVgPA. For participants who need either a much slower or faster music tempo for moderate-intensity PA, they will be arranged into other groups. While twenty participants will be recruited in each residential care facility allocated to the intervention group and based on our previous experiences of pilot study, it is anticipated that three MVgPA groups at most will be conducted in each residential care facility. Participants of the same group will select their music from a list appropriate music, which ranges from children song to pop song, with the required music tempo for the group-based moderate-intensity PA.  Control group  As control group, residential care facilities will conduct their usual activities during the study period. A trained RA will record the activities conducted by the residential care facilities during the study period. If interested, then adults with ID who are assigned to the control group will receive the MVgPA program after all data collection. |
|  | (iii) | Contingency / alternative plan if any problem encountered during implementation |
|  |  | The major problem that may be encountered in the project implementation is the difficulty in recruiting a sufficient number of adults with ID to achieve the targeted sample size. In addition to the collaboration formed in our previous projects, we will continue seeking collaborations from different non-government organizations. Currently, our team has established collaboration with several non-government organizations, including Hon Chi Association, HK Lutheran Social Service, Fu Hong Society and Po Leung Kuk, for the project implementation. The collaborating residential care facilities have agreed to support in participant recruitment and project implementation as stated in the letter of collaboration. In addition, the participants will receive a supermarket coupon (HK$ 50) as compensation for their participation in each phase of the project to enhance the response and intervention completion rate, as well as the completions of follow-ups.  Stepping is a relatively safe PA. A cue card will be provided to the staff of residential care facilities to remind them to seek immediate medical assistance if their service users experience health/medical emergencies during the project implementation. |
|  | (iv) | Cross-sector collaboration |
|  |  | As mentioned before, the organizational capacity of the residential facilities plays an important role in promoting PA of adults with ID^4^. Apart from providing support on participant recruitment and venue for MVgPA, the residential service providers will be asked during the preparation of interventional material about their opinions on the selection guide and the program itself. This project also aims to enhance the knowledge and skill of the staff about PA promotion by supporting them to act as facilitators of the program and by incorporating the intervention into their daily activities. |
|  | (v) | Indicators and targets |
|  |  | The reach-effectiveness-adoption-implementation-maintenance (RE-AIM) framework will be used to evaluate the acceptability and effectiveness of the intervention.  ***Reach*** will be measured by the proportion of eligible adults with ID who have received and completed the intervention relative to the number of adults with ID who have been approached to participate in the program, which will be recorded during the project.  ***Effectiveness*** will be measured by determining whether the following are observed among participants in the intervention group, compared to those in the control, after the intervention:   - a greater increment of at least 0.35 standardized mean difference (SMD) in mean PA level as measured by their time spent in moderate-to-vigorous PA on the basis of empirical evidence^22^; - a greater increment of at least 0.5 SMD in mean level of PA self-efficacy and interest; and - a greater increment of at least 0.5 SMD in mean functional exercise capacity as measured by conducting a 6MWT.   A 0.5 SMD is equivalent to a medium effect size and is conventionally accepted as clinically relevant difference. The satisfaction level of the participants and the staff will also be determined to assess the acceptability of the intervention.  ***Adoption*** will be measured by the proportion of potential residential care facilities that agreed to promote and implement our intervention relative to all facilities that have been approached, which will be recorded during the project.  ***Implementation*** will be measured by the number of intervention sessions that have been delivered at the residential care facility and the number of participants in each session. Attendance will be taken at each intervention session held at each residential care facility.  ***Maintenance*** will be measured by the proportion of residential care facilities allocated to intervention group that have expressed their willingness to continue implementing the intervention at their centres at post-intervention and the number of MVgPA sessions conducted in a 3-month follow-up period. |
|  | (vi) | Evaluation plan |
|  |  | This project will adopt a cluster-randomized controlled group design to examine the effectiveness of the MVgPA.  **Data collection procedures**  Written consent will be obtained from the participants and their parent or guardian before the data collection. The PA level, PA self-efficacy and interest, and functional exercise capacity of the participants will be measured at the baseline (T0), one week post-intervention (T1) and three months post-intervention (T2) by a trained research assistant (RA2) who is blinded to the group allocation. Information on the sociodemographic and clinical characteristics of the participants will be collected by means of a demographic data sheet via interview and reviewing their record. In addition, the PA organized by the residential care facilities during the study period will also be recorded.  **Outcome measurement**  The PA level of participants (primary outcome) will be assessed in terms of minutes of moderate-to-vigorous PA by using an activity tracker (Fitbit Charge 2), which has demonstrated validity as compared with a research-grade accelerometer, and has been used extensively in western and local studies of adults with ID^9,27,28^. The PA level will be calculated as the average time (minutes) of moderate-to-vigorous PA after five consecutive weekdays of measurement at T0, T1 and T2, as they might go back home during the weekend. The staff of residential care facilities will be asked to remind participants to wear the activity tracker during all waking hours except in wet environment, such as showering, and continue with their usual daily activities during the measurement. The staff of residential care facilities will also be asked to help participants to record the time and reason that the activity tracker is put on and taken off each day.  PA self-efficacy will be measured by using the Chinese version of the self-efficacy scale of the baseline interview questionnaire (BIQ-C)^15^, which includes five items pertaining to the confidence of an individual in performing exercise. The items are rated on a three-point Likert scale (1=not at all sure; 3 = totally sure). The BIQ-C has demonstrated acceptable reliability (Cronbach’s ɑ = 0.75) and construct validity among the Chinese adults with ID^15^.  The functional exercise capacity of the participants will be assessed by conducting 6MWT, which has shown to be reliable and valid when used on adults with ID^29^. The participants will be asked to walk as quickly as possible on a flat, hard surface along a straight path in a period of 6 min.  The interest in performing PA and satisfaction level of MVgPA program of participants will be assessed by using a visual analogue scale (VAS), which consists of a 100 mm long line with the left anchor representing “no interest/not satisfied at all” and the right anchor representing “most interested/most satisfied”.  The satisfaction of residential service providers with the MVgPA intervention and their willingness to continue with the program will be measured at T1 by using an author-developed questionnaire. The staff involved in the MVgPA will be asked to rate their satisfaction and willingness on a six-point Likert scale.  **Ethical considerations**  Ethical approval will be sought from the research ethics committee of the study institution. Participants and their parents/guardians will be assured of confidentiality of any data collected, their participation is voluntary and of their right to withdraw from the study at any time without giving a reason, which will not affect their treatment or relationship with their service providers and the university. |
|  | (vii) | Results analysis |
|  |  | Appropriate descriptive statistics will be used to summarize and present the study data. Skewed continuous variables will be appropriately transformed before analysis. By considering the potential design effect of the randomization conducted in cluster level instead of individual participants, the outcome analysis will be performed on the basis of a three-level mixed effects model accounting for inter-correlation among individuals within the same cluster as well as intra-correlation over time within an individual and variations between individuals. This type of model can account for intra-correlated clustered and repeated measures data and produce unbiased estimates even in the presence of missing data, provided that the data are missing at random. Mixed-effects model will be used to compare the differential changes in each outcome variables (PA level, PA self-efficacy and interest, and exercise capacity) across time (T0, T1, T2) between the two groups. Positive significant group-by-T1 and group-by-T2 interaction-terms in the mixed-effects models indicate significantly greater increment of intervention group in the underlying outcome than the control group at the respective time-points with respect to T0.  The primary analysis of the outcome comparisons will be conducted on the basis of intention-to-treat principle, That means all the randomized participants will be included in the analyses and analyzed according to the groups they are originally allocated. Since all the baseline data, including outcomes, will be collected from all participants before randomization, so all the participants should have at least one measurement (observation) for each outcome and can be included in the mixed-effects model for outcome comparison. For participants who could not follow the intervention owing to the change in medical conditions or other reasons, their data will be continued to be collected at the follow-up time points. If there are considerable missing follow-up data, say >10%, sensitivity analysis will also be conducted to assess the robustness of the primary analysis results. In this connection, multiple imputation method will be used to impute missing data, using all available baseline characteristics as covariates. Predictive mean matching approach^30^ which relies less on the parametric assumptions of the imputation models will be used. Furthermore, a post hoc subgroup analysis will be performed to examine the effects of the intervention between those with and those without Down syndrome if a considerable proportion of the participants have such syndrome. Cohen’s d effect sizes will be computed for the outcomes to demonstrate the effectiveness of the programme. All statistical analyses will be conducted using SAS release 9.4 (SAS Institute Inc, Cary, NC). All statistical tests involved will be two-sided with level of significance set at 0.05. |
|  |  | |
|  | Key References: | |
|  | 1. Wong CW. Adults with intellectual disabilities living in Hong Kong’s residential care facilities: A descriptive analysis of health and disease patterns by sex, age, and presence of down syndrome. *J Policy Pract Intellect Disabil.*. 2011;8(4):231-238. 2. Census and Statistics Department, Hong Kong SAR. *Hong Kong monthly digest of statistics: Persons with disabilities and chronic diseases in Hong Kong.* Hong Kong SAR: Census and Statistics Department, Hong Kong SAR; 2015. 3. Fujiura GT, Li H, Magaña S. Health services use and costs for Americans with intellectual and developmental disabilities: A national analysis. *Intellect Dev Disabil*. 2018;56(2):101-118. 4. O'Leary L, Taggart L, Cousins W. Healthy lifestyle behaviours for people with intellectual disabilities: An exploration of organizational barriers and enablers. *J Appl Res Intellect Disabil.* 2018;31:122-135. 5. Blondell SJ, Hammersley-Mather R, Veerman JL. Does physical activity prevent cognitive decline and dementia?: A systematic review and meta-analysis of longitudinal studies. *BMC Public Health*. 2014;14(1):510. 6. Shin I, Park E. Meta-analysis of the effect of exercise programs for individuals with intellectual disabilities. *Res Dev Disabil*. 2012;33(6):1937-1947. 7. Dairo YM, Collett J, Dawes H, Oskrochi GR. Physical activity levels in adults with intellectual disabilities: A systematic review. *Preventive Medicine Reports*. 2016;4:209-219. 8. Center for Health Protection, The Department of Health, HKSAR. *Behavioral risk factor survey.* Hong Kong SAR: Department of Health, HKSAR; 2017. 9. Chow BC, Choi PHN, Hung WYJ. Physical activity and physical fitness of adults with intellectual disabilities in groups homes in Hong Kong. *Intl J Environ Res Public Health*, 2018. doi:10.3390/ijerph150713770. 10. Bossink LW, van der Putten, Annette AJ, Vlaskamp C. Understanding low levels of physical activity in people with intellectual disabilities: A systematic review to identify barriers and facilitators. *Res Dev Disabil*. 2017;68:95-110. 11. Stancliffe RJ, Anderson LL. Factors associated with meeting physical activity guidelines by adults with intellectual and developmental disabilities. *Res Dev Disabil*. 2017;62:1-14. 12. Brooker K, Van Dooren K, McPherson L, Lennox N, Ware R. Systematic review of interventions aiming to improve involvement in physical activity among adults with intellectual disability. *J Phys Act Health* 2015;12(3):434-444. 13. Willems M, Hilgenkamp TI, Havik E, Waninge A, Melville CA. Use of behaviour change techniques in lifestyle change interventions for people with intellectual disabilities: A systematic review. *Res Dev Disabil*. 2017;60:256-268. 14. DiMatteo MR, Haskard-Zolnierek KB, Martin LR. Improving patient adherence: A three-factor model to guide practice. *Health Psychol Rev*. 2012;6(1):74-91. 15. Chan A. Psychosocial and physical activity behavior among adults with intellectual disabilities in Hong Kong. *Br J Arts Socl Sci*, 2012:11(1); 86 - 95. 16. Dixon‐Ibarra A, Driver S, Nery‐Hurwit M, VanVolkenburg H. Qualitative evaluation of a physical activity health promotion programme for people with intellectual disabilities in a group home setting. *J Appl Res Intellect Disabil.*  2018;31:97-109. 17. Martínez-Aldao D, Martínez-Lemos I, Bouzas-Rico S, Ayán-Pérez C. Feasibility of a dance and exercise with music programme on adults with intellectual disability. *J Intellect Disabil Res* 2019. Doi:10.111/ijr.12585. 18. Karageorghis CI, Priest D. Music in exercise domain: A review and synthesis (Part I). Int Rev Sport Exerc Psychol. 2012:5(1); 44-66. 19. Hutzler Y, Korsensky O. Motivational correlates of physical activity in persons with an intellectual disability: A systematic literature review. *J Intellect Disabil Res*. 2010:54(9);767-789. 20. Obrusnikova I, Firkin CJ, Farquhar WB. A systematic review and meta-analysis of the effects of aerobic exercise interventions on cardiorespiratory fitness in adults with intellectual disability. *Disabil Health J.* 2022;15(1):101185. doi: 10.1016/j.dhjo.2021.101185. 21. Adams G, Gulliford MC, Ukoumunne OC, Eldridge S, Chinn S, Campbell MJ. Patterns of intra-cluster correlation from primary care research to inform study design and analysis. *J Clin Epidemiol*. 2004;57(8):785-794. 22. American College of Sports Medicine. *ACSM’s guidelines for exercise testing and prescription (10^th^ ed.).* Baltimore, MD: Wolters Kluwer/Lippincott Williams & Wilkins Health; 2018. 23. Finlayson J, Turner A, Granat MH. Measuring the actual levels and pattern of physical activity/inactivity of adults with intellectual disabilities. J Applied Res Intellect Disabil. 2011;24:508-517. 24. Tudor-Locke ,. Rowe DA. Using cadence to study free-living ambulatory behavior. Sport Med. 2012;42(5):381-398. 25. Rehabilitation Advisory Committee, Labour and Welfare Bureau (2016). Reports of the working group on ageing of persons with intellectual disabilities under the Rehabilitation Advisory Committee. Retrieved from: https://www.lwb.gov.hk/eng/other_info/Report%20of%20Working%20Group%20on%20Ageing%20of%20PwIDs%202016_c.pdf 26. Shigematsu R, Okura T, Nakagaichi M, et al. Square-stepping exercise and fall risk factors in older adults: A single-blind, randomized controlled trial. *J Gerontol A Biol Sci Med Sci.* 2008;63(1):76-82. 27. Brewer W, Swanson BT, Ortiz A. Validity of fitbit’s active minutes as compared with a research-grade accelerometer and self-reported measures. *BMJ Open Sport Exerc Med*. 2017;3(1):e000254. 28. Philips AC, Holland AJ. Assessment of objectively measured physical activity levels in individual with intellectual disabilities with and without Down’s Syndrome. *PLoS One*. 2011. Doi:10.1371/journal.pone.0028618. 29. Nasuti G, Stuart-Hill L, Temple VA. The six-minute walk test for adults with intellectual disability: A study of validity and reliability. *J Intellect Dev Disabil.* 2013;38(1):31-38. 30. Little RJA. Missing-data adjustments in large surveys. J Business Econ Stat. 1988;6:287- 296. | |

**Appendix 1. Pre-participation health screening for individual starting with a new physical activity program^1-2^**

Participants will be screened for: (1) individual’s current level of physical activity (i.e. performing planned, structured physical activity at least 30 minutes at moderate intensity on at least 3 days/week for at least 3 months; (2) presence of signs and symptoms of cardiovascular, metabolic or renal disease, and (3) desired exercise intensity.

To ensure participants’ safety to participate in the MVgPA, pre-participation health screening based on the American College of Sports Medicine Preparticipation Screening algorithm^1^ will be used.

| **Known cardiovascular, metabolic or renal disease** | - Cardiac, peripheral vascular or cerebrovascular disease - Diabetes (Type I or II) - Renal disease | Approval form a health care professional or medical clearance to engage in exercise is needed |
| --- | --- | --- |
| **Signs or symptoms suggestive of cardiovascular, renal or metabolic disease (at rest or during activity):** | - Pain, discomfort in the chest, neck, jaw, arms or other areas that may result from ischemia - Shortness of breath at rest or with mild exertion - Dizziness or syncope - Orthopnea or paroxysmal nocturnal dyspnea - Ankle edema - Palpitations or tachycardia - Intermittent claudication - Known heart murmur - Unusual fatigue or shortness of breath with usual activities |  |

**Note**

^1^American College of Sports Medicine. ACSM’s guidelines for exercise testing and prescription. 10th ed. Philadelphia: Wolters Kluwer/Lippincott Williams & Wilkins Health; 2018.

^2^Center for Health Protection, Department of Health, Hong Kong SAR. Non-communicable disease watch: March 2016. Hong Kong SAR: Department of Health; 2016.

**Appendix 2. Conceptual underpinning of MVgPA**

**Appendix 3. Intervention plan for the MVgPA**

| **Wk** | **Intervener/**  **format** | **IMS model** | **Strategies adopted** | |
| --- | --- | --- | --- | --- |
| 1 | **IMS for staff:**  **Preparatory session**  RN and RA  90-min group discussion  ***(increase organizational capacity)*** | Information | Health talk on ways to achieve adequate PA, its associated health benefits, and the safety concerns related to moderate-intensity PA | |
|  |  | Motivation | Identify three positive outcomes of the MVgPA program on their service users for setting goals of the MVgPA sessions | |
|  |  | Strategy | Roll out resistance of PA implementation   - Determine three most significant obstacles - Devise specific solutions for the identified obstacle in with the RN   Practice the MVgPA with the RN and RA. | |
|  | **12-week music–visual guided PA session for adults with ID**  (75 min/session; two session/week) | Information, motivation & strategy | - 75-min music–visual guided physical activities will be presented in the PowerPoint slideshow (including stepping, upper limb exercise with peer interaction, and warm-up and cool-down exercise targeting on stretching the major muscle–tendon units of shoulder, body trunk and limbs before and after) facilitated by RA and/or trained staff. - In 10-min bout of MVgPA   - oriented about the health benefit of adequate PA   - Verbal persuasion that physical activity as achievable (e.g., we can do it!) | |
| 2 | Two 75-min sessions led by RA1 and co-facilitated by the staff of supported care setting. | | | **Ongoing support to staff:**  The first six session will be led by RA1 and co-facilitate by the staff to support the staff to conducted the subsequent sessions. |
| 3 | Two 75-min sessions led by RA1 and co-facilitated by the staff of supported care setting. | | |  |
| 4 | Two 75-min sessions led by RA1 and co-facilitated by the staff of supported care setting. | | |  |
| 5 | *Two 75-min sessions led by staff of supported care setting* | | | |
| 6 | **Motivation:** Implementation of a new set of music-paced physical activities   - Two 75-min music-paced physical activities led by RA1 and co-facilitated by the staff of supported care setting. | | | **Motivation & strategy**   - The new set of music-paced physical activities will be led by RA1 and co-facilitated by the staff. - Meeting with staff   - Acknowledge the achievement of the staff and of MVgPA   - Address concerns, identify facilitators and tackle barriers related to the MVgPA implementation. |
| 7 - 8 | *Two 75-min sessions led by staff of supported care setting* | | | |
| 9 | *Two 75-min music-paced physical activities led by staff of supported care setting* | | | **Motivation & strategy**   - Meeting with staff   - Acknowledge the achievement of the staff and of MVgPA   - Address concerns, identify facilitators and tackle barriers related to the MVgPA implementation. |
| 10-12 | *Two 75-min music-paced physical activities led by staff of supported care setting* | | | |
