# Supplementary material for: Effects of a music-visual guided physical activity promotion program for adults with intellectual disability in supported care settings: a cluster-randomized controlled trial
Source: Int J Behav Nutr Phys Act. 2026 Jan 17;23:11. doi: 10.1186/s12966-026-01872-6 (PMC12896249; doi:10.1186/s12966-026-01872-6)
Supplement: Supplementary file 3 — Supplementary Material 3. [file 12966_2026_1872_MOESM3_ESM.docx]

**Supplementary Table 1 Pre-participation health screening for individual starting with a new physical activity program^1-3^**

Participants will be screened for: (1) individual’s current level of physical activity (i.e. performing planned, structured physical activity at least 30 minutes at moderate intensity on at least 3 days/week for at least 3 months; (2) presence of signs and symptoms of cardiovascular, metabolic or renal disease, and (3) desired exercise intensity.

| **Known cardiovascular, metabolic or renal disease** | - Cardiac, peripheral vascular or cerebrovascular disease - Diabetes (Type I or II) - Renal disease | Approval form a health care professional to engaged in exercise is needed;  Exercise is suggested to taken place in a facility with a medically qualified staff.  Exclude from the current study. |
| --- | --- | --- |
| **Signs or symptoms suggestive of cardiovascular, renal or metabolic disease (at rest or during activity):** | - Pain, discomfort in the chest, neck, jaw, arms or other areas that may result from ischemia - Shortness of breath at rest or with mild exertion - Dizziness or syncope - Orthopnea or paroxysmal nocturnal dyspnea - Ankle edema - Palpitations or tachycardia - Intermittent claudication - Known heart murmur - Unusual fatigue or shortness of breath with usual activities |  |

**Note**

^1^American College of Sports Medicine. ACSM’s guidelines for exercise testing and prescription. 9th ed. Philadelphia: Wolters Kluwer/Lippincott Williams & Wilkins Health; 2014.

^2^Riebe D, Franklin BA, Thompson PD, Garber CE, Whitfield GP, Magal M, et al. Updating ACSM’s recommendations for exercise participation health screening. Med Sci Sports Exerc 2015;47(8):2473-2479.

^3^Center for Health Protection, Department of Health, Hong Kong SAR. Non-communicable disease watch: March 2016. Hong Kong SAR: Department of Health; 2016.

**Supplementary Figure 1. Conceptual underpinning of MVgPA**

**Supplementary Table 2. Intervention plan for the MVgPA**

| **Wk** | **Intervener/**  **format** | **IMS model** | **Strategies adopted** | |
| --- | --- | --- | --- | --- |
| 1 | **IMS for staff:**  **Preparatory session**  RN and RA  90-min group discussion  ***(increase organizational capacity)*** | Information | Health talk on ways to achieve adequate PA, its associated health benefits, and the safety concerns related to moderate-intensity PA | |
|  |  | Motivation | Identify three positive outcomes of the MVgPA program on their service users for setting goals of the MVgPA sessions | |
|  |  | Strategy | Roll out resistance of PA implementation   - Determine three most significant obstacles - Devise specific solutions for the identified obstacle in with the RN   Practice the MVgPA with the RN and RA. | |
|  | **12-week music–visual guided PA session for adults with ID**  (75 min/session; two session/week) | Information, motivation & strategy | - 75-min music–visual guided physical activities will be presented in the PowerPoint slideshow (including stepping, upper limb exercise with peer interaction, and warm-up and cool-down exercise targeting on stretching the major muscle–tendon units of shoulder, body trunk and limbs before and after) facilitated by RA and/or trained staff. - In 10-min bout of MVgPA   - oriented about the health benefit of adequate PA   - Verbal persuasion that physical activity as achievable (e.g., we can do it!) | |
| 2 | Two 75-min sessions led by RA1 and co-facilitated by the staff of supported care setting. | | | **Ongoing support to staff:**  The first six session will be led by RA1 and co-facilitate by the staff to support the staff to conducted the subsequent sessions. |
| 3 | Two 75-min sessions led by RA1 and co-facilitated by the staff of supported care setting. | | |  |
| 4 | Two 75-min sessions led by RA1 and co-facilitated by the staff of supported care setting. | | |  |
| 5 | *Two 75-min sessions led by staff of supported care setting* | | | |
| 6 | **Motivation:** Implementation of a new set of music-paced physical activities   - Two 75-min music-paced physical activities led by RA1 and co-facilitated by the staff of supported care setting. | | | **Motivation & strategy**   - The new set of music-paced physical activities will be led by RA1 and co-facilitated by the staff. - Meeting with staff   - Acknowledge the achievement of the staff and of MVgPA   - Address concerns, identify facilitators and tackle barriers related to the MVgPA implementation. |
| 7 - 8 | *Two 75-min sessions led by staff of supported care setting* | | | |
| 9 | *Two 75-min music-paced physical activities led by staff of supported care setting* | | | **Motivation & strategy**   - Meeting with staff   - Acknowledge the achievement of the staff and of MVgPA   - Address concerns, identify facilitators and tackle barriers related to the MVgPA implementation. |
| 10-12 | *Two 75-min music-paced physical activities led by staff of supported care setting* | | | |
